# Association of maternal history of neonatal death with subsequent neonatal death across 56 low- and middle-income countries

**DOI:** 10.1038/s41598-021-97481-3

**Published:** 2021-10-07

**Authors:** Zhihui Li, Mudit Kapoor, Rockli Kim, S. V. Subramanian

**Affiliations:** 1 Vanke School of Public Health, Tsinghua University, Beijing, China; 2 Economics and Planning Unit, Indian Statistical Institute (ISI), New Delhi, India; 3 Division of Health Policy and Management, College of Health Sciences, Korea University, Seoul, 02841 South Korea; 4 Interdisciplinary Program in Precision Public Health, Department of Public Health Sciences, Graduate School of Korea University, Seoul, South Korea; 5 Harvard Center for Population & Development Studies, 9 Bow Street, Cambridge, MA USA; 6 Department of Social and Behavioral Sciences, Harvard T.H. Chan School of Public Health, Boston, MA USA

**Keywords:** Risk factors, Epidemiology

## Abstract

Early identification of high-risk pregnancies can reduce global neonatal mortality rate. Using the most recent Demographic and Health Surveys from 56 low- and middle-income countries, we examined the proportion of mothers with history of neonatal deaths. Logistic regression models were used to assess the association between maternal history of neonatal death and subsequent neonatal mortality. The adjusted models controlled for socioeconomic, child, and pregnancy-related factors. Country-specific analyses were performed to assess heterogeneity in this association across countries. Among the 437,049 live births included in the study, 6910 resulted in neonatal deaths. In general, 22.4% (1549) occurred to mothers with previous history of neonatal death; at the country-level, this proportion ranged from 1.2% (95% confidence interval [CI] 0.0, 2.6) in Dominican Republic to 38.1% (95% CI 26.0, 50.1) in Niger. Maternal history of neonatal death was significantly associated with subsequent neonatal death in both the pooled and the subgroup analyses. In the fully adjusted model, history of neonatal death was associated with 2.1 (95% CI 1.9, 2.4) times higher odds of subsequent neonatal mortality in the pooled analysis. We observed large variation in the associations across countries ranging from fully adjusted odds ratio (FAOR) of 0.4 (95% CI 0.0, 4.0) in Dominican Republic to 16.1 (95% CI 3.6, 42.0) in South Africa. Our study suggests that maternal history of neonatal death could be an effective early identifier of high-risk pregnancies in resource-poor countries. However, country-specific contexts must be considered in national policy discussions.

## Introduction

The neonatal period (birth to 28 completed days of life) is known as the most critical time for child survival^1^. During the Millennium Development Goal (MDG) era, decline in neonatal deaths was much slower than the decline in under-five mortality rate. Globally, the number of neonatal deaths has reduced by 39% from 4.3 to 2.8 million between 1990 and 2015; while the number of post-neonatal and childhood deaths (after 28 days to 5 years old) has declined by 61% from 7.7 to 3.3 million during this period^2^. The Sustainable Development Goal (SDG) aims to reduce neonatal mortality rate (NMR) to less than 12 per 1000 live births by 2030^3^. To reduce preventable neonatal deaths in the SDG era, it is critical to identify high-risk pregnancies. A rich volume of studies have identified various risk factors of neonatal mortality, including low socioeconomic status, insufficient antenatal care, and low birth weight^4–6^. Yet, only a few studies have assessed the potential importance of maternal history of poor pregnancy outcome. According to our review (up to the date of September 22nd, 2020), only eight studies, which were all at country- or sub country-levels, considered poor pregnancy outcomes as a risk factor of subsequent neonatal deaths; six of the eight studies were low- and middle-income countries, including India, Ethiopia, Bangladesh, and Ghana^7–14^. Previous studies have consistently found maternal history of neonatal deaths to be a significant factor associated with subsequent neonatal deaths^9–14^.

All the prior studies were conducted at national or sub-national levels with insufficient information to reveal whether this association is heterogeneous across countries. The identification of cross-country heterogeneity is essential for the design of global and national health agenda. If the association between history of neonatal deaths and subsequent neonatal mortality turns out to be consistent across countries, then history of neonatal death could be considered as one of the indicators to identify high risk pregnancies in LMICs^10^. On the other hand, if the association shows substantial heterogeneity across countries, then the implication would be to develop more localized guidelines to promote positive pregnancy.

In this paper, we pooled nationally representative data of Demographic and Health Surveys (DHS) from 56 countries to systematically assess the association between maternal history of neonatal death and risk of subsequent neonatal death. In addition to the pooled analysis, we present country-specific findings to inform the extent of heterogeneity across countries.

## Methods

This project used publicly-accessible secondary data requested and downloaded from the DHS website (https://dhsprogram.com/data/available-datasets.cfm). The DHS data are not collected specifically for this study and no one on the study team has access to identifiers linked to the data. These activities do not meet the regulatory definition of human subjects research. As such, IRB review is not required. The Harvard Longwood Campus IRB allows researchers to self-determine when their research does not meet the requirements for IRB oversight via guidance online regarding when an IRB application is required and the IRB Decision Tool.

### Data source and study population

We pooled the most recent data for LMICs conducted between 2010 and 2018 from DHS, which collected detailed information on complete birth history, child information, and household characteristics from 15 to 49 years old women^15^. DHS used a multistage stratified sampling design, with the first stage generally involving choosing geographically-defined units such as villages for rural areas and census blocks for urban areas, and the second stage involving selecting the specific households or persons to be interviewed^16^. We excluded earlier survey rounds to avoid inconsistencies in the measurements, collection, and reporting of data required for this study.

There were 59 LMICs that had collected data on the history of neonatal deaths and subsequent neonatal mortality. We excluded Albania, Armenia, and Turkey from our analysis because the DHS data of these countries recorded less than 10 neonatal deaths within 5 years prior to the survey. The final sample included 56 LMICs. See Appendix Table 1 for a detailed list of countries and survey years included in the study.

There were a total of 602,587 live births recorded in the 56 LMICs. We excluded 156,978 live births from nulliparous women and 8560 nonsingleton live births. A total of 437,049 singleton live births from multiparous women aged 15 to 49 years were included in our analysis. Among all the live births included in the study, 6910 died during the neonatal period.

### History of neonatal death

The primary predictor was maternal history of neonatal death. We identified these cases by examining the complete birth history of the mothers. A binary variable was constructed with the value of 1 representing a previous live birth that resulted in death during the first 28 completed days of life, and 0 otherwise.

### Outcomes

Our main outcome was subsequent neonatal mortality. Following the World Health Organization (WHO) definition, neonatal death refers to the “deaths among live births during the first 28 completed days of life”^17^. For additional analyses, we define early neonatal death as “death among live births between 0 and 7 completed days of birth” and late neonatal death as “death among live births after 7 days to 28 completed days of birth” as our secondary outcomes^18^.

### Covariates

In the adjusted analysis, we followed previous studies^7–14^ and controlled for a comprehensive set of covariates that are known to be associated with neonatal mortality, including socioeconomic and household factors, child factors, and pregnancy-related characteristics. The socioeconomic and household factors included household wealth quintiles, place of residence, maternal education, improved water, improved sanitation, and number of household members. Household wealth quintile was constructed by the DHS based on a selected set of household assets^19^. Place of residence was a dichotomous variable, which categorized the population into urban and rural. We classified maternal education to six categories (no schooling, < 5 years, 5–7 years, 8–9 years, 10–11 years, and 12 years or more)^10^. Water source was considered to be improved if the household had access to water piped into dwelling or yard/plot, public tap/standpipe, tube well or borehole, protected well or spring, rain water, and bottled water^20^. We considered sanitation facility to be improved if the household had access to flush to piped sewer system, septic tank, or pit latrine, ventilated improved pit latrine, pit latrine with slab, and composting toilet^20^. We divided number of household members to three categories (< 6, 6–10, 11 and more). The child factors included sex of the child, birth weight of the child by type of report, size at birth, and breastfeeding initiation. We generated five categories for child birth weight by type of report, which were (1) not weighted, (2) < 2500 g based on mother’s recall, (3) < 2500 g based on written card, (4) $\ge$ 2500 g based on mother’s recall, and (5) $\ge$ 2500 g based on written card^10^. DHS classified child size at birth into three categories: (1) within reference range or higher, (2) small, (3) very small^21^. We generated a dichotomous variable to see whether breastfeeding was initiated < 1 h of birth^22^. The pregnancy-related covariates included number of antenatal cares, full tetanus protection, whether delivered with a skilled birth attendant (SBA), whether delivered in a health facility, maternal age at birth, and birth interval^10^. Global guidelines recommend frequent medical visits during the antenatal period to decrease the risk of birth complications, with recommendation ranging from at least four to optimally eight visits during the pregnancy^23^. We classified the number of antenatal cares into four categories (i.e. 0 times, 1–4 times, 5–7 times, 8 times or more). Maternal age at birth was classified in three categories (< 18, 18–34, 35 years or older) because both young and old maternal age are associated with higher risk of neonatal mortality^24^. Similarly, birth interval was classified in three categories (< 18, 18–59, 60 months and more) because long inter-pregnancy intervals (possibly longer than 5 years) and short intervals are independently associated with adverse pregnancy outcomes^25^. All other pregnancy-related covariates were dichotomized. For observations with missing data on one or more covariates we adopted the MI commands in STATA for multiple imputations^26–28^.

### Statistical analysis

First, we examined the characteristics of the sample by their history of neonatal death. Second, we assessed the proportions of mothers with history of neonatal deaths among all multiparous women with previous live births in each of the 56 LMICs. To calculate the proportion, we performed crude logistic regression without any covariates. Third, we quantified the proportion of all neonatal deaths that have occurred to mothers with history of neonatal deaths using unadjusted logistic regression model.

To assess the association between mothers with history of neonatal death and subsequent neonatal mortality, we performed both pooled analysis and separate analysis for each country. We included sampling weight, clustering, and stratification variables provided by DHS to ensure that the estimates were representative at national level and in pooled analyses^29^. We clustered the sample at PSU level, which allows for interdependence of error terms within clusters and households^29^. In pooled analyses, we reweighted observations by country’s population size, and included country-fixed effects to account for the unobservable country-level factors.

For both pooled and country-specific analyses, we developed three sets of logistic models to assess the association between maternal history of neonatal death and subsequent neonatal mortality. First, we ran a crude logistic model without controlling for any covariate to obtain unadjusted odds ratio (UOR) for subsequent neonatal mortality. Second, we ran a set of logistic models controlling each covariate separately to obtain partially adjusted odds ratio (PAOR). Third, we controlled for all the covariates introduced above to obtain fully adjusted odds ratio (FAOR). We calculated the degree to which OR attenuated after covariate adjustment using the formula $\frac{UOR-PAOR}{UOR-1}*100$ or $\frac{UOR-FAOR}{UOR-1}*100$^30^.

We tested for consistency in the association by performing a series of stratified analyses defined by household wealth, place of residence, maternal education, sex of child, birth weight by report type, birth size, numbers of antenatal care visits, delivery with SBA, institutional delivery, full tetanus protection, and maternal age at birth. We also conducted two sets of supplementary analysis. First, we examined the association between maternal history of neonatal death with subsequent early neonatal mortality and late neonatal mortality, respectively. Second, we divided the history of neonatal death further into history of multiple neonatal deaths (more than one) and history of one neonatal death, and compared the effects between these two groups. We ran both unadjusted and adjusted logistic regressions for the supplementary analyses.

We used Stata (version 14.2) for all analyses procedures. All statistical tests were two-sided and p < 0.05 was considered to determine statistical significance.

## Results

### Descriptive summary

Of the 437,049 singleton live births included in the analysis, 37,290 (8.5%) live births were by mothers with history of neonatal deaths. The NMR among mothers with history of neonatal death was 42.1 (95% confidence interval [CI]: 39.6, 44.6) per 1000 live births, which was higher than those without history of neonatal deaths (13.2 per 1000 live births, 95% CI 12.8, 13.7). Compared to mothers without history of neonatal deaths, mothers with such history were more likely to live in poorer households, in rural areas, with lower education, with babies unweighted or weighted low at birth, have small babies, have less numbers of antenatal care visits, deliver babies without a SBA, deliver babies at home, give births at older ages, have shorter birth intervals, without improved sanitation facility, without improved water access, initiate breastfeeding after 1 h of birth, and have more household members (Table 1).

**Table 1:** Characteristics of the multiparous women stratified by history of neonatal death.

Characteristics	History of neonatal death (percentage or 95% CI)		
	Yes (n = 37,290)	No (n = 399,759)	P value
Neonatal mortality per 1000 live births	42.1 (39.6, 44.6)	13.2 (12.8, 13.7)	< 0.001
**Wealth, quintile**			
Poorest	12,239 (32.8%)	104,507 (25.9%)	< 0.001
Poorer	9009 (24.2%)	91,198 (22.6%)	
Middle	7058 (18.9%)	79,983 (19.8%)	
Richer	5546 (14.9%)	70,300 (17.4%)	
Richest	3438 (9.2%)	58,010 (14.4%)	
**Place of residence**			
Urban	8546 (22.9%)	122,672 (30.7%)	< 0.001
Rural	28,744 (77.1%)	277,087 (69.3%)	
**Maternal education, y**			
0	18,339 (49.2%)	139,550 (34.9%)	< 0.001
< 5	4372 (11.7%)	42,876 (10.7%)	
5–7	6229 (16.7%)	74,132 (18.5%)	
8–9	3326 (8.9%)	48,505 (12.1%)	
10–11	1839 (4.9%)	37,466 (9.4%)	
≥ 12	2260 (6.1%)	49,368 (12.4%)	
Missing	925 (2.5%)	7862 (2.0%)	
**Sex of child**			
Male	19,096 (51.2%)	208,651 (52.2%)	0.02
Female	18,194 (48.8%)	191,108 (47.8%)	
**Birth weight**			
Not weighted	14,875 (39.9%)	122,203 (30.6%)	< 0.001
Low			
Mother's recall	1595 (4.3%)	14,988 (3.8%)	
Written card	1090 (2.9%)	11,202 (2.8%)	
Within reference range or higher			
Mother's recall	8518 (22.8%)	110,875 (27.7%)	
Written card	7829 (21.0%)	99,798 (25.0%)	
Missing	3383 (9.1%)	40,693 (10.2%)	
**Birth size**			
Within reference range or higher	29,172 (78.2%)	318,505 (79.7%)	< 0.001
Small	4527 (12.1%)	40,049 (10.0%)	
Very small	1997 (5.4%)	14,988 (3.8%)	
Missing	1594 (4.3%)	26,217 (6.6%)	
**Antenatal care visits, no**			
0	6931 (18.6%)	61,171 (15.3%)	< 0.001
1–4	18,525 (49.7%)	180,712 (45.2%)	
5–7	7643 (20.5%)	93,458 (23.4%)	
≥ 8	3707 (9.9%)	58,795 (14.7%)	
Missing	484 (1.3%)	5623 (1.4%)	
**Delivered with skilled birth attendant**			
Yes	18,743 (50.3%)	230,425 (57.6%)	< 0.001
No	18,494 (49.6%)	169,021 (42.3%)	
Missing	53 (0.1%)	313 (0.1%)	
**Institutional delivery**			
Yes	21,953 (58.9%)	266,231 (66.6%)	< 0.001
No	15,337 (41.1%)	133,528 (33.4%)	
**Full tetanus protection**			
Yes	27,082 (72.6%)	290,386 (72.6%)	0.02
No	10,208 (27.4%)	109,373 (27.4%)	
**Maternal age at birth, y**			
< 18	310 (0.8%)	3447 (0.9%)	< 0.001
18–34	27,486 (73.7%)	332,008 (83.1%)	
≥ 35	9494 (25.5%)	64,304 (16.1%)	
**Birth interval, mo**			
< 18	5342 (14.3%)	25,893 (6.5%)	< 0.001
18–59	27,116 (72.7%)	296,804 (74.3%)	
≥ 60	4832 (13.0%)	77,062 (19.3%)	
**Improved sanitation**			
Yes	14,200 (38.1%)	194,851 (48.7%)	< 0.001
No	21,103 (56.6%)	184,093 (46.1%)	
Missing	1987 (5.3%)	20,815 (5.2%)	
**Improved water**			
Yes	23,733 (63.6%)	261,898 (65.5%)	< 0.001
No	23,733 (29.7%)	110,623 (27.7%)	
Missing	2479 (6.7%)	27,238 (6.8%)	
**Breastfeeding initiation < 1 h of birth**			
Yes	16,043 (43.0%)	191,803 (48.0%)	< 0.001
No	18,577 (49.8%)	180,600 (45.2%)	
Missing	2670 (7.2%)	27,356 (6.8%)	
**Number of household members**			
< 6	14,072 (37.7%)	164,684 (41.2%)	< 0.001
6–10	18,420 (49.4%)	189,339 (47.4%)	
≥ 11	4798 (12.9%)	45,736 (11.4%)	

**Figure 1:**
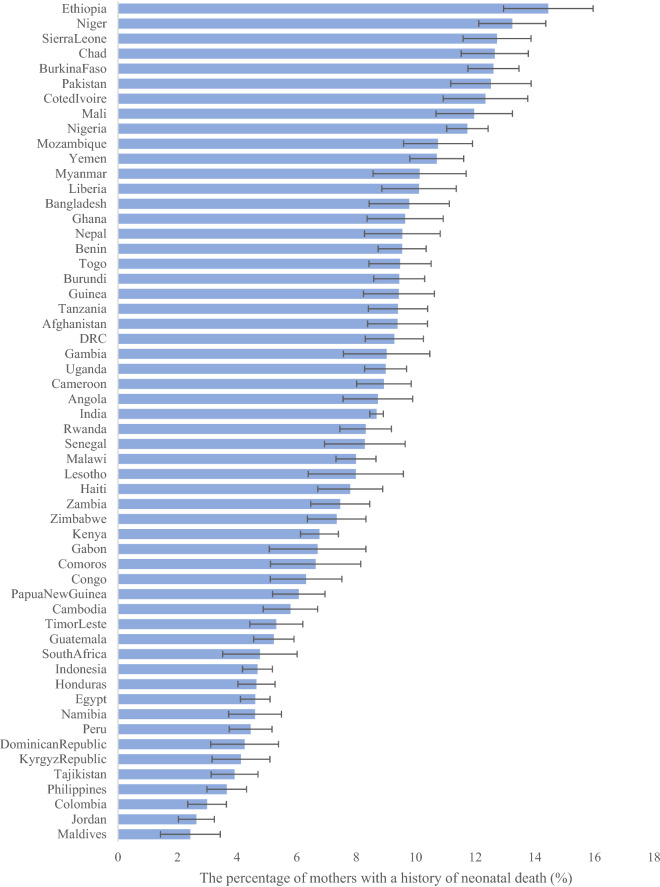
Between-country variations in percent of mothers with history of neonatal death.

The proportion of multiparous women with history of neonatal death varied across countries: only 2.4% (95% CI 1.4, 3.4) of the multiparous women had a previous history of neonatal death in Maldives, followed by Jordan (2.6%, 95% CI 2.0, 3.2), and Colombia (3.0%, 95% CI 2.3, 3.6). Countries with the highest proportions were Ethiopia (14.5%, 95% CI 12.9, 16.0), Niger (13.2%, 95% CI 12.1, 14.4), and Sierra Leone (12.7%, 95% CI 11.6, 13.9) (Fig. 1).

**Figure 2:**
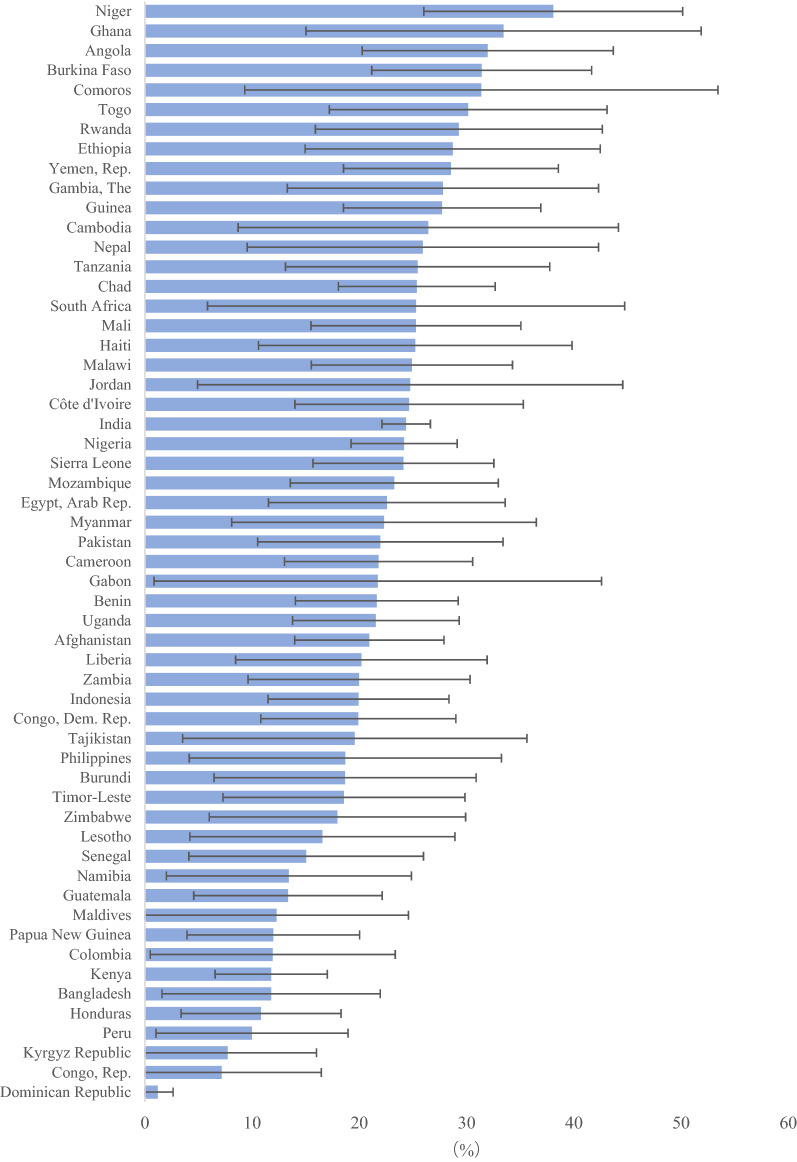
The percentage of neonatal deaths occurring to mothers with history of neonatal death.

For all 6910 neonatal deaths recorded among the most recent live births, 1549 (22.4%) occurred to mothers with previous history of neonatal deaths. Countries with the lowest proportions were Dominican Republic (1.2%, 95% CI 0.0, 2.6), Congo, Rep. (7.1%, 95% CI 0.0, 16.4), and Kyrgyz Republic (7.7%, 95% CI 0.0, 16.0); while countries with the highest proportions were Niger (38.1%, 95% CI 26.0, 50.1), Ghana (33.4%, 95% CI 15.0, 51.9), and Angola (32.0%, 95% CI 20.3, 43.7) (Fig. 2).

### Pooled analyses

In our pooled analysis, we found a strong association between maternal history of neonatal death and subsequent neonatal mortality, albeit FAOR attenuated by 50.7% compared to UOR. In the fully adjusted logistic regression models, history of neonatal death was associated with 2.1 (95% CI 1.9, 2.4) higher odds of subsequent neonatal mortality, and this remained consistent across different subgroups with only one exception – for the subgroup of very small babies at birth, although history of neonatal death was significantly associated with higher odds of subsequent neonatal mortality in the unadjusted model (UOR = 2.25, 95% CI 1.75, 2.88); after covariate adjustment, FAOR was estimated to be 1.00 (95%: 0.64, 1.57), which was not statistically significant (Table 2).

**Table 2:** Unadjusted odds ratio (UOR) and fully adjusted odds ratios (FAOR) for neonatal mortality by maternal history of neonatal death, pooled sample and subgroup samples.

Variable	UOR (95% CI)	FAOR (95% CI)	Change (%)
Overall	3.25 (3.03, 3.50)	2.11 (1.86, 2.40)	50.7
**Wealth, quintile**			
Poorest	3.08 (2.71, 3.49)	2.36 (1.90, 2.93)	34.6
Poorer	3.16 (2.74, 3.63)	1.89 (1.48, 2.41)	58.8
Middle	3.36 (2.84, 3.96)	2.17 (1.63, 2.88)	50.4
Richer	3.11 (2.58, 3.75)	1.66 (1.18, 2.33)	68.7
Richest	3.50 (2.74, 4.46)	2.18 (1.26, 3.78)	52.8
**Place of residence**			
Urban	3.44 (2.95, 4.01)	1.85 (1.31, 2.62)	65.2
Rural	3.17 (2.92, 3.44)	2.17 (1.89, 2.49)	46.1
**Maternal education, y**			
0	2.86 (2.58, 3.17)	2.24 (1.90, 2.65)	33.3
< 5	2.86 (2.58, 3.17)	2.24 (1.59, 3.15)	33.3
5–7	3.49 (2.85, 4.27)	1.49 (1.06, 2.10)	80.3
8–9	2.94 (2.45, 3.52)	2.00 (1.30, 3.08)	48.5
10–11	3.24 (2.55, 4.12)	2.58 (1.23, 5.42)	29.5
≥ 12	3.81 (2.74, 5.29)	2.39 (1.21, 4.72)	50.5
**Sex of child**			
Male	3.23 (2.93, 3.56)	1.97 (1.65, 2.36)	56.5
Female	3.32 (2.98, 3.69)	2.28 (1.90, 2.73)	44.8
**Birth weight**			
Not weighted	2.84 (2.55, 3.15)	2.21 (1.83, 2.65)	34.2
Low			
Mother's recall	2.23 (1.71, 2.92)	1.47 (1.09, 2.06)	94.3
Written card	2.08 (1.20, 3.61)	1.63 (1.01, 2.67)	85.2
Within reference range or higher			
Mother's recall	3.32 (2.81, 3.93)	2.45 (1.87, 3.21)	37.5
Written card	3.47 (2.65, 4.56)	2.16 (1.44, 3.26)	53.0
**Birth size**			
Within reference range or higher	3.37 (3.10, 3.68)	2.37 (2.06, 2.74)	42.2
Small	2.48 (2.04, 3.01)	1.65 (1.13, 2.40)	56.1
Very small	2.25 (1.75, 2.88)	1.00 (0.64, 1.57)	100.0
**Antenatal care visits, no**			
0	4.14 (3.08, 5.55)	2.01 (1.58, 2.56)	67.8
1–4	2.96 (2.54, 3.46)	2.17 (1.81, 2.61)	40.3
5–7	3.01 (2.71, 3.34)	1.85 (1.36, 2.53)	57.7
≥ 8	3.38 (2.88, 3.98)	2.80 (1.73, 4.55)	24.4
**Delivered with skilled birth attendant**			
Yes	3.82 (2.98, 4.90)	2.03 (1.70, 2.43)	63.5
No	3.44 (3.12, 3.80)	2.19 (1.82, 2.62)	51.2
**Institutional delivery**			
Yes	3.01 (2.70, 3.36)	2.19 (1.85, 2.60)	40.8
No	3.44 (3.13, 3.77)	2.01 (1.66, 2.44)	58.6
**Full tetanus protection**			
Yes	2.89 (2.57, 3.24)	2.16 (1.85, 2.51)	38.6
No	3.24 (2.97, 3.53)	2.02 (1.60, 2.56)	54.5
**Maternal age at birth, y**			
< 18	3.27 (2.87, 3.73)	2.02 (2.02, 2.02)	55.1
18–34	3.26 (2.99, 3.55)	2.05 (1.76, 2.40)	53.5
≥ 35	2.71 (2.38, 3.10)	2.29 (1.82, 2.88)	24.6
**Birth interval, mo**			
< 18	3.15 (2.66, 3.73)	1.92 (1.45, 2.54)	57.2
18–59	3.11 (2.85, 3.39)	2.25 (1.93, 2.63)	40.8
≥ 60	2.60 (2.13, 3.19)	1.60 (1.06, 2.42)	62.5
**Improved sanitation**			
Yes	2.84 (2.58, 3.12)	2.04 (1.75, 2.39)	43.5
No	3.63 (3.22, 4.08)	2.23 (1.78, 2.80)	53.2
**Improved water**			
Yes	2.90 (2.53, 3.32)	1.93 (1.54, 2.43)	51.1
No	3.44 (3.15, 3.76)	2.20 (1.89, 2.56)	50.8
**Breastfeeding initiation < 1 h of birth**			
Yes	2.57 (2.18, 3.01)	1.96 (1.66, 2.33)	38.9
No	3.05 (2.58, 3.62)	2.28 (1.88, 2.76)	37.6
**Number of household members**			
< 6	3.43 (3.10, 3.79)	1.87 (1.55, 2.25)	64.2
6–10	3.06 (2.73, 3.44)	2.16 (1.76, 2.65)	43.7
≥ 11	3.84 (3.07, 4.79)	3.13 (2.20, 4.44)	25.0

(1) “Change” is the degree to which the odds ratios were attenuated after adjustment that was calculated according to the formula $\frac{UOR-FAOR}{UOR-1}*100$.

When compared to estimates from partially adjusted models, the timing of breastfeeding initiation had the largest attenuation effect, causing the coefficient of history of neonatal death to reduce by 20.9% from an UOR: 3.25 (95% CI 3.03, 3.50) to a PAOR: 2.78 (95% CI 2.47, 3.13) (Appendix Table 2). This was followed by covariates on birth interval, birth weight and maternal age at birth, which attenuated the UOR by 6–10% each.

### Country-specific analyses

**Figure 3:**
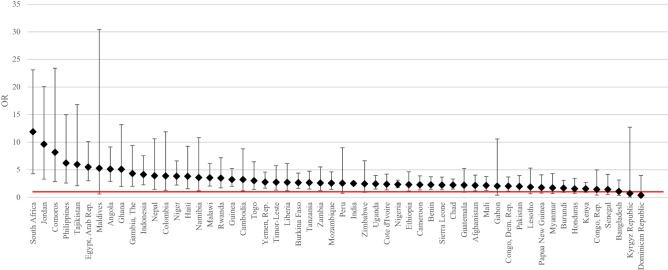
Fully adjusted odds ratios (FAOR) for neonatal mortality by maternal history of neonatal death, by country.

The unadjusted and fully adjusted associations between history of neonatal death and subsequent neonatal mortality for each country are presented in Fig. 3 and Appendix Table 3. The FAOR varied from 0.4 (95% CI 0.0, 4.0) in Dominican Republic to 16.1 (95% CI 3.6, 42.0) in South Africa. We found 40 of the 56 countries with statistically significant association between history of neonatal death and subsequent neonatal mortality. The top five countries with the largest FAOR were South Africa, Egypt (11.7, 95% CI 4.5, 39.7), Jordan (10.7, 95% CI 3.5, 32.9), Philippines (6.2, 95% CI 2.6, 15.0), and Tajikistan (6.0, 95% CI 2.1, 16.8). There were 16 countries with statistically insignificant association between history of neonatal death and subsequent neonatal mortality using the adjusted model (e.g. Bangladesh, Burundi, Gabon). (1) We fully adjusted the models with factors related to socioeconomic environment, maternal anthropometry, and pregnancy care. (2) We excluded Albania and Armenia as they have less than 20 observations with recorded maternal history of neonatal death. (3) Bangladesh, Kyrgyz Republic, Tajikistan, and Turkey do not have valid data on full tetanus protection. Therefore, we excluded this covariate from the model. (4) Yemen does not have valid data on maternal education. Therefore, we excluded this covariate from the model.

The FAORs attenuated by more than 10% in 39 of the 56 countries compared to their respective UORs, with the largest attenuation occurring in Kyrgyz Republic (change: 128.1%, UOR: 2.0, FAOR: 0.7), followed by Comoros (change: 80.0%, UOR: 6.9, FAOR: 2.2) and Gabon (change: 64.7%, UOR: 4.0; FAOR: 2.1) (Appendix Table 3).

### Supplementary analysis

We performed two sets of supplementary analysis. First, we examined whether history of neonatal death had a stronger association with subsequent neonatal mortality in the earlier period (i.e. 0 and 7 completed days of birth) than in the later period (i.e. after 7 days to 28 days of birth). With the pooled sample, we found the history of neonatal death to be associated with 2.7 (95% CI 2.5, 3.0) higher odds of subsequent early neonatal death using the fully adjusted model, which was larger than the association for subsequent late neonatal death (FAOR: 1.8, 95% CI 1.5, 2.2) (Appendix Figure 1). Second, we investigated whether history of multiple neonatal death has a stronger association than history of single neonatal death. We found that the association was FAOR: 2.8 (95% CI 2.3, 3.5) among those with multiple previous neonatal deaths, and FAOR: 1.9 (95% CI 1.7, 2.3) for those with only one previous neonatal death (Appendix Figure 2).

In the country-level analysis, we found that among the 42 countries with sufficient observations (more than 20 deaths) for both early neonatal and late neonatal periods, there were 25 countries with the associations being stronger for subsequent early neonatal mortality than for subsequent late neonatal mortality. For example, in Gambia, the history of neonatal deaths was associated with 4.6 (95% CI 2.1, 10.2) times higher odds of subsequent early neonatal death, which was greater than that for subsequent late neonatal death at 0.7 (95% CI 0.1, 4.0) (Appendix Table 4). Furthermore, 39 of the 50 countries with sufficient observations had stronger associations among mothers with multiple previous neonatal deaths than those with only one previous neonatal death. The difference between the two groups reached statistically significance (p < 0.01) in 9 countries, including Bangladesh, Egypt, Haiti, India, Kenya, Mali, Nepal, South Africa, and Togo (Appendix Table 5).

## Discussion

Four salient findings emerged from our analysis using the most recent data from 56 LMICs. First, the proportion of mothers with history of neonatal death varied widely across countries from 2 to 15%. We also found substantial cross-country variation in the proportion of neonatal deaths that has occurred to mothers with history of neonatal death, ranging from 1 to 38%. Second, we found maternal history of neonatal deaths to be strongly associated with subsequent neonatal deaths in both pooled and a series of subgroup analyses, even after adjusting for a comprehensive set of covariates. Third, at the country-level, we found the associations between maternal history of neonatal death and subsequent neonatal death to vary largely across countries. The FAOR ranged between 0.4 and 16.1, yet the majority of countries had statistically significant association. The attenuation between UOR and FAOR was the largest in Kyrgyz Republic, Comoros, and Gabon. Lastly, the association between history of neonatal deaths and subsequent neonatal mortality was stronger in the earlier neonatal periods (i.e. 0–7 completed days) in both the pooled and most country-level analyses. Moreover, the association was stronger among mothers with multiple previous neonatal deaths than those with one previous neonatal death.

The large variation in the proportions of mothers with history of neonatal death suggests that with appropriate interventions, the prevalence of history of neonatal deaths in many countries can be potentially modified. For example, in Maldives, only 2% of the women had one or more previous neonatal deaths, and this is closely related to the country’s continuous efforts to tackle maternal and child health in the past two decades. Health workers in Maldives received additional training and were deployed to provide home visits and close monitoring, particularly for women with high-risk pregnancies. Health officials, along with international organizations (e.g. World Health Organization, United Nations Population Fund, etc.), expanded emergency obstetric care availability and family planning access to ensure all pregnancies were wanted and were taken proper care of^31,32^. The Health Master Plan for 2006–2015 put a strong focus on the newborn health in Maldives, which was further strengthened by the Maldives Every Newborn Action Plan (2014) with a commitment to end all preventable newborn deaths^32^. As a result, NMR in Maldives reduced by 89% from 42 per 1000 live births in 1990 to 5 in 2016^33^.

Our study also found a wide variation in the proportions of neonatal deaths that have occurred to mothers with one or more previous neonatal deaths. In Niger, among all newborns who died within 28 completed days of birth, almost two fifths of them happened to mothers with history of neonatal death. Two reasons could partially explain this striking variation. First, since a larger proportion of the Nigerien mothers (13%) experienced death of newborns before, it was more likely for a child to be born to these mothers. Second, other risk factors, such as poor socioeconomic status, obstetric and pediatric resources available to the households, and the related health behavior during antenatal and intrapartum periods, are likely to persist and constantly affect pregnancies at different times^9–14^. However, it is notable that even after we adjusted for a rich set of covariates, the association remained statistically significant. Similarly, we found that despite large attenuation, the association between maternal history of neonatal deaths and subsequent neonatal mortality remained strong in many countries, including Burkina Faso, Jordan, and Timor-Leste. This suggests that there might be either a direct causal link between the history of neonatal death and subsequent neonatal deaths or an indirect link via other unobservable residual confounding related to environmental factors, function of health care system, quality of care, and genetic, immunological, or etiological disorders^10,34,35^.

Dominican Republic, Congo, Rep., and Kyrgyz Republic had less than 10% of the newborn deaths occurred to mothers with previous neonatal deaths, and the associations between previous neonatal deaths and subsequent newborn mortality were also weak. This may be a result of the countries’ existing efforts to identify high-risk pregnancies. For example, in Kyrgyz Republic, attempts were made to ensure women’s uptake of timely obstetric care from specialists, including the Promotion of Perinatal Health project that specially aimed to strengthen maternal and newborn referral system^36^. In Dominican Republic, community health workers have been trained to perform essential roles in identifying pregnancy complications and being responsible to report high risk patients to the doctors^37,38^.

Despite such scattered efforts in several countries, in general, most LMICs still lack specific programs or interventions to identify mothers with high-risk pregnancies who would benefit the most from intensive obstetric and pediatric surveillance and care during antenatal, intrapartum, and postpartum periods^35,39–41^. Maternal history of neonatal death, as a simple identification tool for high-risk pregnancy, can enhance timely referral, effective maternal counselling, early diagnosis and treatment of specific disorders, and access to higher-level health facilities for obstetric and pediatrician care^35^.

There are several limitations to this study. First, we were only able to include 56 LMICs with available data. Therefore, the estimates from our pooled sample were not representative at either global-level or by income groups. Second, we were unable to include previous stillbirth as part of the covariates because this indicator was not collected or specified as a separate category in majority of the countries. Third, the usage of observational data and cross-sectional analysis limit our capacity to make any causal inference. Although we have adjusted for numerous socioeconomic, child, and pregnancy-related factors, our analyses are still subject to confounders that are unobserved or without available data. There were some potential confounders that were not collected in DHS or with substantial amount of missing values, such as the quality of delivery care, the usage of insecticide-treated bed nets, and pregnancy complications. Fourth, despite the fact that we limited our data to the most recent surveys conducted in a relatively small range of time (between 2010 and 2018), the survey year of each country varied from each other which might be a challenge for a cross-sectional study. Last, although DHS has been widely adopted as the most reliable source on child mortality^42–45^, we recognize the potential data collection problems, including misreporting dates to birth or age at death^46^.

Using the most comprehensive and up-to-date data, our investigation indicates that adverse outcomes of previous pregnancies is a strong risk factor of future neonatal deaths in a majority of the studied countries. This finding suggests that maternal history of neonatal deaths could be a powerful and effective indicator to identify women with high-risk pregnancies, especially in LMICs. At the same time, the substantial heterogeneity across countries indicates the need for context-specific understanding to inform national policies and programs. Therefore, we call for more national and regional level studies for the design of localized interventions to identify high-risk pregnancies and reduce neonatal mortality.

## Supplementary Information

Supplementary Information.
