# Supplementary material for: Association of maternal history of neonatal death with subsequent neonatal death across 56 low- and middle-income countries
Source: Sci Rep. 2021 Oct 7;11:19919. doi: 10.1038/s41598-021-97481-3 (PMC8497561; doi:10.1038/s41598-021-97481-3)
Supplement: Supplementary file 1 — Supplementary Information. [file 41598_2021_97481_MOESM1_ESM.docx]

**Supplementary Annex:**

Association of Maternal History of Neonatal Death with Subsequent Neonatal Death across 56 Low- and Middle-Income Countries

**Appendix Table 1. Countries and survey years involved in the study**

| **Country** | **Survey year** | **Income class** |
| --- | --- | --- |
| Afghanistan | 2015 | L |
| Angola | 2015-16 | UM |
| Bangladesh | 2014 | LM |
| Benin | 2017-18 | L |
| Burkina Faso | 2010 | L |
| Burundi | 2016-17 | L |
| Cambodia | 2014 | L |
| Cameroon | 2018 | LM |
| Chad | 2014-15 | L |
| Colombia | 2015 | UM |
| Comoros | 2012 | L |
| Congo, Rep. | 2011-12 | LM |
| Cote d'Ivoire | 2011-12 | LM |
| Congo, Dem. Rep. | 2013-14 | L |
| Dominican Republic | 2013 | UM |
| Egypt, Arab Rep. | 2014 | LM |
| Ethiopia | 2016 | L |
| Gabon | 2012 | UM |
| Gambia, The | 2013 | L |
| Ghana | 2014 | LM |
| Guatemala | 2014-15 | LM |
| Guinea | 2018 | L |
| Haiti | 2016-17 | L |
| Honduras | 2011-12 | LM |
| India | 2015-16 | LM |
| Indonesia | 2017 | LM |
| Jordan | 2017-18 | UM |
| Kenya | 2014 | LM |
| Kyrgyz Republic | 2012 | L |
| Lesotho | 2014 | LM |
| Liberia | 2013 | L |
| Malawi | 2015-16 | L |
| Maldives | 2016-17 | UM |
| Mali | 2018 | L |
| Mozambique | 2011 | L |
| Myanmar | 2015-16 | LM |
| Namibia | 2013 | UM |
| Nepal | 2016 | L |
| Niger | 2012 | L |
| Nigeria | 2018 | LM |
| Pakistan | 2017-18 | LM |
| Papua New Guinea | 2016-18 | LM |
| Peru | 2012 | UM |
| Philippines | 2017 | LM |
| Rwanda | 2014-15 | L |
| Senegal | 2018 | LM |
| Sierra Leone | 2013 | L |
| South Africa | 2016 | UM |
| Tajikistan | 2017 | L |
| Tanzania | 2015-16 | L |
| Timor-Leste | 2016 | LM |
| Togo | 2013-14 | L |
| Uganda | 2016 | L |
| Yemen | 2013 | LM |
| Zambia | 2018 | LM |
| Zimbabwe | 2015 | L |

Note:

“L” represents “low-income countries”; “LM” represents “lower-middle-income countries”; “UM” represents “upper-middle-income countries”.

**Appendix Table 2. The attenuation effect between unadjusted (UOR) and partially adjusted odds ratios (PAOR) for neonatal mortality by maternal history of neonatal death, pooled sample**

| **Covariates** | **PAOR (95% CI)** | **Change (%)** | |  |  |
| --- | --- | --- | --- | --- | --- |
| **Wealth, quintile** |  |  | |  |  |
| Poorest | 3.23(3.00, 3.47) | 1.0% | |  |  |
| Poorer | 3.25(3.02, 3.49) | 0.1% | |  |  |
| Middle | 3.25(3.03, 3.50) | -0.1% | |  |  |
| Richer | 3.24(3.02, 3.49) | 0.2% | |  |  |
| Richest | 3.22(3.00, 3.47) | 1.2% | |  |  |
| **Place of residence, urban** | 3.23(3.00, 3.47) | 0.9% | |  |  |
| **Maternal education, y** | | |  | |  |
| 0 | 3.14(2.92, 3.38) | 4.8% | |  |  |
| <5 | 3.25(3.02, 3.50) | -0.1% | |  |  |
| 5-7 | 3.25(3.02, 3.50) | 0.0% | |  |  |
| 8-9 | 3.24(3.02, 3.49) | 0.3% | |  |  |
| 10-11 | 3.22(2.99, 3.46) | 1.4% | |  |  |
| ≥12 | 3.20(2.98, 3.44) | 2.2% | |  |  |
| **Sex of child, male** | 3.26(3.04, 3.51) | -0.7% | |  |  |
| **Birth weight** |  |  | |  |  |
| Not weighted | 3.05(2.83, 3.28) | 9.0% | |  |  |
| Low |  |  | |  |  |
| Mother's recall | 3.24(3.01, 3.48) | 0.5% | |  |  |
| Written card | 3.25(3.03, 3.50) | -0.2% | |  |  |
| Within reference range or higher | | |  | |  |
| Mother's recall | 3.20(2.97, 3.44) | 2.4% | |  |  |
| Written card | 3.11(2.89, 3.35) | 6.1% | |  |  |
| **Birth size** |  |  | |  |  |
| Within reference range or higher | 3.22(2.99, 3.46) | 1.6% | |  |  |
| Small | 3.23(3.01, 3.48) | 0.8% | |  |  |
| Very small | 3.18(2.95, 3.42) | 3.3% | |  |  |
| **Antenatal care visits, No**. | | |  | |  |
| 0 | 3.18(2.96, 3.43) | 2.9% | |  |  |
| 1-4 | 3.26(3.03, 3.51) | -0.6% | |  |  |
| 5-7 | 3.24(3.01, 3.48) | 0.5% | |  |  |
| 8-9 | 3.22(2.99, 3.46) | 1.5% | |  |  |
| ≥10 | 3.23(3.00, 3.47) | 1.0% | |  |  |
| **Delivered with skilled birth attendant** | 3.23(3.00, 3.48) | 0.9% | |  |  |
| **Institutional delivery** | 3.19(2.97, 3.44) | 2.5% | |  |  |
| **Full tetanus protection** | 3.25(3.02, 3.50) | 0.0% | |  |  |
| **Maternal age at birth, y** | | |  | |  |
| <18 | 3.25(3.03, 3.50) | -0.2% | | | |
| 18-34 | 3.11(2.89, 3.34) | 6.3% | | | |
| ≥35 | 3.10(2.89, 3.34) | 6.5% | | | |
| **Birth interval, mo** |  |  | | | |
| <18 | 3.04(2.82, 3.27) | 9.6% | | | |
| 18-59 | 3.24(3.02, 3.49) | 0.5% | | | |
| ≥59 | 3.25(3.02, 3.49) | 0.2% | | | |
| **Improved sanitation** | 3.12(2.90, 3.37) | 5.7% | | | |
| **Improved water** | 3.27(3.04, 3.52) | -1.0% | | | |
| **Breastfeeding initiation <1 hour of birth** | 2.78(2.47, 3.13) | 20.9% | | | |
| **Number of household members** |  |  | | | |
| <6 | 3.32(3.09, 3.57) | -3.3% | | | |
| 6-10 | 3.29(3.06, 3.54) | -1.8% | | | |
| ≥10 | 3.26(3.03, 3.51) | -0.5% | | | |

Note:

1. “Change” is the degree to which the odds ratios were attenuated after adjustment that was calculated according to the formula $\frac{UOR-PAOR}{UOR-1}*100$

**Appendix Table 3. Unadjusted (UOR) and fully adjusted odds ratios (FAOR) for neonatal mortality by maternal history of neonatal death**

| **Country** | **UOR** | **FAOR** | **Change** |
| --- | --- | --- | --- |
| Afghanistan | 2.6(1.6, 4.2) | 2.2(1.2, 4.0) | 26.5% |
| Angola | 5.2(3.0, 8.9) | 5.1(2.9, 9.1) | 1.7% |
| Bangladesh | 1.2(0.5, 3.2) | 1.1(0.4, 3.1) | 52.7% |
| Benin | 2.7(1.7, 4.2) | 2.3(1.4, 3.7) | 24.5% |
| Burkina Faso | 3.2(2.0, 5.2) | 2.7(1.6, 4.4) | 23.7% |
| Burundi | 2.2(1.0, 4.9) | 1.7(0.9, 3.1) | 42.5% |
| Cambodia | 6.2(2.4, 15.8) | 3.2(1.2, 8.8) | 57.2% |
| Cameroon | 2.9(1.8, 4.9) | 2.3(1.3, 3.9) | 32.0% |
| Chad | 2.4(1.6, 3.6) | 2.2(1.5, 3.3) | 11.6% |
| Colombia | 4.5(1.5, 13.5) | 3.9(1.3, 11.9) | 17.6% |
| Comoros | 6.9(2.7, 17.9) | 2.2(1.9, 3.4) | 80.0% |
| Congo, Dem. Rep. | 2.5(1.4, 4.4) | 2.0(1.1, 3.7) | 30.5% |
| Congo, Rep. | 1.1(0.3, 4.7) | 1.4(0.4, 5.0) | -344.5% |
| Cote d'Ivoire | 2.4(1.3, 4.2) | 2.4(1.4, 4.2) | 0.5% |
| Dominican Republic | 0.3(0.0, 2.1) | 0.4(0.0, 4.0) | 12.9% |
| Egypt, Arab Rep. | 6.2(3.3, 11.6) | 11.7(4.5, 39.7) | -106.5% |
| Ethiopia | 2.4(1.3, 4.8) | 2.3(1.1, 4.6) | 6.7% |
| Gabon | 4.0(1.2, 12.9) | 2.1(0.4, 10.6) | 64.7% |
| Gambia, The | 4.0(1.9, 8.3) | 4.3(2.0, 9.4) | -11.2% |
| Ghana | 4.9(2.1, 11.2) | 5.1(2.0, 13.2) | -5.2% |
| Guatemala | 2.8(1.3, 6.2) | 2.2(0.9, 5.2) | 32.5% |
| Guinea | 3.9(2.4, 6.1) | 3.2(2.0, 5.2) | 23.0% |
| Haiti | 4.2(1.9, 9.2) | 3.8(1.6, 9.3) | 11.9% |
| Honduras | 2.5(1.2, 5.5) | 1.6(0.7, 3.4) | 61.6% |
| India | 3.5(3.1, 4.0) | 2.5(2.2, 2.9) | 39.0% |
| Indonesia | 5.3(3.1, 9.0) | 4.1(2.3, 7.6) | 26.9% |
| Jordan | 13.2(4.4, 39.0) | 10.7(3.5, 32.9) | 20.5% |
| Kenya | 1.9(1.1, 3.1) | 1.6(0.9, 2.7) | 36.3% |
| Kyrgyz Republic | 2.0(0.4, 9.6) | 0.7(0.0, 12.7) | 128.1% |
| Lesotho | 2.4(1.0, 5.8) | 1.9(0.7, 5.3) | 37.5% |
| Liberia | 2.3(1.1, 4.8) | 2.7(1.2, 6.1) | -32.4% |
| Malawi | 3.9(2.3, 6.6) | 3.6(2.0, 6.2) | 11.7% |
| Maldives | 5.8(1.4, 24.6) | 5.3(0.6, 30.4) | 10.7% |
| Mali | 2.6(1.5, 4.4) | 2.2(1.2, 3.8) | 27.1% |
| Mozambique | 2.6(1.5, 4.4) | 2.6(1.4, 4.6) | 0.8% |
| Myanmar | 2.6(1.1, 6.0) | 1.7(0.7, 4.3) | 54.2% |
| Namibia | 3.3(1.3, 8.7) | 3.6(1.2, 10.8) | -13.1% |
| Nepal | 3.4(1.4, 8.0) | 3.9(1.4, 10.6) | -21.4% |
| Niger | 4.1(2.5, 6.9) | 3.8(2.2, 6.6) | 8.3% |
| Nigeria | 2.5(1.9, 3.2) | 2.3(1.8, 3.1) | 10.4% |
| Pakistan | 2.0(1.0, 4.0) | 2.0(1.0, 4.0) | 0.1% |
| Papua New Guinea | 2.1(1.0, 4.6) | 1.8(0.8, 4.1) | 29.4% |
| Peru | 2.4(0.9, 6.5) | 2.6(0.7, 9.0) | -12.6% |
| Philippines | 6.4(2.4, 17.0) | 6.2(2.6, 15.0) | 3.2% |
| Rwanda | 4.7(2.5, 9.0) | 3.5(1.7, 7.2) | 32.2% |
| Senegal | 2.0(0.8, 4.8) | 1.4(0.5, 4.2) | 56.6% |
| Sierra Leone | 2.2(1.4, 3.5) | 2.2(1.4, 3.7) | -3.3% |
| South Africa | 7.5(2.7, 21.1) | 16.1(3.6, 42.0) | -131.6% |
| Tajikistan | 6.1(2.2, 17.4) | 6.0(2.1, 16.8) | 2.6% |
| Tanzania | 3.4(1.8, 6.5) | 2.7(1.5, 4.7) | 30.4% |
| Timor-Leste | 4.2(2.0, 9.1) | 2.7(1.3, 5.8) | 45.4% |
| Togo | 4.3(2.3, 7.9) | 3.0(1.4, 6.5) | 38.6% |
| Uganda | 2.9(1.8, 4.6) | 2.5(1.5, 4.0) | 23.6% |
| Yemen, Rep. | 3.4(2.1, 5.6) | 2.8(1.7, 4.6) | 26.2% |
| Zambia | 3.2(1.6, 6.2) | 2.6(1.2, 5.5) | 27.0% |
| Zimbabwe | 2.8(1.2, 6.5) | 2.5(0.9, 6.6) | 18.8% |

Note:

1. We marked the insignificant associations in red
2. “Change” is the degree to which the odds ratios were attenuated after adjustment that was calculated according to the formula $\frac{UOR-FAOR}{UOR-1}*100$

**Appendix Figure 1. Unadjusted (UOR) and fully adjusted odds ratio (FAOR) for period of occurrence of mortality stratified by maternal history of neonatal death**

1. **UOR**

3.46 (3.20, 3.75)

2.33 (1.96, 2.78)

1. **FAOR**

2.74 (2.52, 2.98)

1.82 (1.51, 2.19)

Note: The two ORs in both unadjusted and adjusted models were significant different from each other with p<0.001

**Appendix Figure 2. Unadjusted (UOR) and fully adjusted odds ratios (FAOR) for neonatal mortality stratified by maternal history of one neonatal death or more than one neonatal deaths**

1. **UOR**

2.84 (2.62, 3.09)

5.28 (4.68, 5.97)

1. **FAOR**

2.81 (2.28, 3.47)

1.94 (1.68, 2.25)

Note: The two ORs in both unadjusted and adjusted models were significant different from each other with p<0.001

**Appendix Table 4. Fully adjusted odds ratio for period of occurrence of mortality stratified by maternal history of neonatal death by country**

| **Country** | **Early neonatal death** | **Late neonatal death** | **Difference** | |
| --- | --- | --- | --- | --- |
|  |  |  | **P value** | **Direction** |
| Afghanistan | 2.69(1.47, 4.92) | 1.11(0.42, 2.94) | 0.000 | + |
| Angola | 5.52(3.01, 10.11) | 2.02(0.37, 11.03) | 0.000 | + |
| Bangladesh | 1.17(0.27, 5.15) | 0.89(0.16, 5.00) | 0.133 | Insignificant |
| Benin | 2.37(1.43, 3.94) | 1.15(0.27, 4.92) | 0.000 | + |
| Burkina Faso | 2.61(1.43, 4.78) | 2.66(1.19, 5.91) | 0.087 | Insignificant |
| Burundi | 1.56(0.72, 3.36) | 1.75(0.66, 4.65) | 0.077 | Insignificant |
| Cambodia | 3.14(0.88, 11.19) | 3.47(0.90, 13.34) | 0.054 | Insignificant |
| Cameroon | 2.24(1.19, 4.22) | 2.84(0.76, 10.56) | 0.000 | - |
| Chad | 2.69(1.72, 4.20) | 1.17(0.49, 2.79) | 0.000 | + |
| Colombia | 6.58(1.95, 22.21) | 0.69(0.07, 7.10) | 0.108 | Insignificant |
| Congo, Dem. Rep. | 2.04(1.07, 3.90) | 2.01(0.49, 8.21) | 0.054 | Insignificant |
| Cote d'Ivoire | 2.94(1.62, 5.34) | 0.74(0.18, 3.04) | 0.000 | + |
| Egypt, Arab Rep. | 4.54(1.81, 11.41) | 8.23(2.74, 24.71) | 0.311 | Insignificant |
| Ethiopia | 2.96(1.44, 6.06) | 0.07(0.01, 0.50) | 0.000 | + |
| Gabon | 1.86(0.23, 14.97) | 4.09(0.66, 25.28) | 0.463 | Insignificant |
| Gambia, The | 4.63(2.11, 10.16) | 0.65(0.11, 4.03) | 0.000 | + |
| Guatemala | 2.35(0.86, 6.42) | 2.21(0.56, 8.66) | 0.001 | + |
| Guinea | 3.58(2.14, 5.97) | 1.21(0.13, 11.32) | 0.000 | + |
| Haiti | 5.05(1.61, 15.85) | 2.18(0.62, 7.63) | 0.000 | + |
| India | 2.65(2.31, 3.04) | 1.81(1.30, 2.53) | 0.000 | + |
| Indonesia | 4.11(2.21, 7.63) | 3.14(0.48, 20.67) | 0.000 | + |
| Kenya | 1.84(1.02, 3.29) | 0.58(0.12, 2.77) | 0.000 | + |
| Lesotho | 2.12(0.62, 7.28) | 0.88(0.05, 15.32) | 0.007 | + |
| Liberia | 3.42(1.40, 8.34) | 1.94(0.24, 15.78) | 0.000 | + |
| Malawi | 4.22(2.35, 7.60) | 0.80(0.19, 3.32) | 0.000 | + |
| Mali | 2.66(1.49, 4.76) | 0.49(0.08, 3.06) | 0.000 | + |
| Mozambique | 2.50(1.37, 4.55) | 3.19(0.51, 19.83) | 0.000 | - |
| Nepal | 2.75(0.90, 8.37) | 20.95(3.24, 135.51) | 0.052 | Insignificant |
| Niger | 3.32(1.74, 6.33) | 6.90(2.72, 17.50) | 0.001 | - |
| Nigeria | 2.37(1.74, 3.24) | 2.14(1.09, 4.19) | 0.000 | + |
| Pakistan | 2.36(1.20, 4.63) | 0.81(0.11, 5.75) | 0.000 | + |
| Papua New Guinea | 2.33(0.99, 5.48) | 0.14(0.01, 1.45) | 0.000 | + |
| Philippines | 6.05(2.25, 16.24) | 6.77(1.67, 27.49) | 0.009 | - |
| Rwanda | 4.90(2.32, 10.37) | 0.32(0.05, 1.98) | 0.000 | + |
| Senegal | 1.54(0.50, 4.73) | 1.75(0.14, 22.14) | 0.009 | - |
| Sierra Leone | 2.09(1.21, 3.61) | 3.40(1.21, 9.58) | 0.000 | - |
| Tajikistan | 6.99(2.59, 18.89) | 13.80(1.63, 117.21) | 0.194 | Insignificant |
| Timor-Leste | 3.00(1.39, 6.47) | 1.56(0.00, 598.66) | 0.001 | + |
| Uganda | 2.46(1.42, 4.24) | 2.26(0.94, 5.40) | 0.000 | + |
| Yemen, Rep. | 3.19(1.78, 5.74) | 1.73(0.65, 4.59) | 0.000 | + |
| Zambia | 3.33(1.46, 7.58) | 0.81(0.19, 3.42) | 0.001 | + |
| Zimbabwe | 0.92(0.20, 4.24) | 10.25(2.75, 38.19) | 0.013 | - |

Note:

We excluded the countries with the number of early neonatal deaths or the number of late neonatal deaths less than 20.

“+” means the OR for early neonatal death is larger than that for late neonatal death;

“-” means the OR for early neonatal death is smaller than that for late neonatal death.

**Appendix Table 5. Fully adjusted odds ratio for neonatal mortality stratified by maternal history of one neonatal death or more than one neonatal deaths by country**

| **Country** | **History of more than one neonatal death** | **History of one neonatal death** | **Difference** | |
| --- | --- | --- | --- | --- |
|  |  |  | **P value** | **Direction** |
| Afghanistan | 2.9(1.4, 6.0) | 2.1(1.0, 4.1) | 0.455 | Insignificant |
| Angola | 5.1(1.8, 14.7) | 5.2(2.7, 10.1) | 0.980 | Insignificant |
| Bangladesh | 7.9(1.5, 40.5) | 0.5(0.1, 1.8) | 0.008 | + |
| Benin | 3.0(1.3, 7.3) | 2.1(1.2, 3.7) | 0.468 | Insignificant |
| Burkina Faso | 3.5(1.6, 7.7) | 2.5(1.4, 4.4) | 0.463 | Insignificant |
| Burundi | 2.1(0.6, 7.6) | 1.6(0.8, 3.2) | 0.695 | Insignificant |
| Cambodia | 3.7(0.8, 17.7) | 2.9(0.9, 9.0) | 0.785 | Insignificant |
| Cameroon | 2.5(1.0, 6.4) | 2.2(1.1, 4.4) | 0.835 | Insignificant |
| Chad | 2.3(1.2, 4.5) | 2.2(1.4, 3.5) | 0.921 | Insignificant |
| Comoros | 18.7(3.6, 98.2) | 5.7(1.6, 20.7) | 0.234 | Insignificant |
| Congo, Rep. | 1.6(0.2, 10.1) | 1.4(0.3, 5.8) | 0.935 | Insignificant |
| Cote d'Ivoire | 2.3(0.9, 5.7) | 2.4(1.2, 4.8) | 0.955 | Insignificant |
| Congo, Dem. Rep. | 2.6(0.9, 7.2) | 1.9(1.0, 3.4) | 0.546 | Insignificant |
| Egypt, Arab Rep. | 22.8(7.2, 72.5) | 3.2(1.5, 6.8) | 0.006 | + |
| Ethiopia | 4.0(1.0, 16.3) | 2.2(1.1, 4.7) | 0.413 | Insignificant |
| Gabon | 1.9(0.1, 55.0) | 2.2(0.4, 12.7) | 0.948 | Insignificant |
| Gambia, The | 10.5(2.9, 38.2) | 2.6(1.0, 6.8) | 0.063 | Insignificant |
| Ghana | 12.3(3.2, 47.2) | 3.8(1.3, 11.5) | 0.119 | Insignificant |
| Guatemala | 1.7(0.5, 5.5) | 2.3(0.9, 5.8) | 0.648 | Insignificant |
| Guinea | 4.3(1.8, 10.4) | 2.9(1.7, 5.1) | 0.447 | Insignificant |
| Haiti | 12.5(4.1, 38.2) | 2.2(0.8, 6.4) | 0.018 | + |
| Honduras | 4.1(0.5, 32.0) | 1.3(0.6, 3.0) | 0.317 | Insignificant |
| India | 4.0(3.2, 5.0) | 2.2(1.9, 2.6) | 0.000 | + |
| Indonesia | 2.1(0.3, 14.0) | 4.8(2.6, 8.8) | 0.406 | Insignificant |
| Jordan | 52.8(4.5, 618.2) | 6.5(2.0, 21.5) | 0.119 | Insignificant |
| Kenya | 5.8(2.5, 13.0) | 0.8(0.4, 1.5) | 0.000 | + |
| Liberia | 1.6(0.4, 6.7) | 3.1(1.3, 7.5) | 0.426 | Insignificant |
| Malawi | 5.8(2.1, 15.6) | 3.1(1.7, 5.6) | 0.282 | Insignificant |
| Mali | 4.2(1.9, 9.2) | 1.6(0.8, 3.2) | 0.036 | + |
| Mozambique | 4.0(1.5, 10.3) | 2.2(1.1, 4.2) | 0.261 | Insignificant |
| Myanmar | 2.0(0.4, 9.7) | 1.6(0.6, 4.5) | 0.838 | Insignificant |
| Namibia | 9.7(1.8, 51.6) | 2.9(0.8, 10.8) | 0.256 | Insignificant |
| Nepal | 15.2(3.0, 76.3) | 2.5(0.7, 9.0) | 0.043 | + |
| Niger | 1.8(0.6, 5.6) | 4.8(2.7, 8.2) | 0.095 | Insignificant |
| Nigeria | 2.8(1.8, 4.4) | 2.2(1.6, 3.1) | 0.382 | Insignificant |
| Pakistan | 3.4(1.4, 8.7) | 1.7(0.7, 4.0) | 0.240 | Insignificant |
| Papua New Guinea | 1.5(0.2, 12.7) | 1.7(0.7, 4.3) | 0.918 | Insignificant |
| Peru | 3.5(0.3, 41.1) | 2.3(0.6, 9.3) | 0.768 | Insignificant |
| Philippines | 4.3(0.5, 34.9) | 6.8(2.7, 17.0) | 0.688 | Insignificant |
| Rwanda | 3.0(1.1, 8.4) | 3.6(1.6, 8.2) | 0.757 | Insignificant |
| Senegal | 3.0(0.6, 15.7) | 1.1(0.3, 3.9) | 0.288 | Insignificant |
| Sierra Leone | 2.7(1.2, 5.8) | 2.1(1.2, 3.7) | 0.594 | Insignificant |
| South Africa | 44.3(8.9, 221.7) | 7.6(2.6, 22.4) | 0.050 | + |
| Tajikistan | 6.8(0.3, 174.6) | 5.8(2.0, 17.0) | 0.927 | Insignificant |
| Tanzania | 4.1(1.3, 13.4) | 2.4(1.3, 4.4) | 0.384 | Insignificant |
| Timor-Leste | 5.5(1.1, 26.6) | 2.1(0.9, 4.7) | 0.251 | Insignificant |
| Togo | 9.1(3.2, 26.2) | 1.9(0.8, 4.4) | 0.005 | + |
| Uganda | 4.6(2.0, 10.6) | 2.0(1.2, 3.4) | 0.070 | Insignificant |
| Zambia | 2.7(0.9, 8.0) | 2.5(1.1, 6.0) | 0.942 | Insignificant |
| Zimbabwe | 1.9(0.2, 19.0) | 2.6(0.9, 7.3) | 0.809 | Insignificant |

Note:

We excluded the countries with the number of one previous neonatal death or the number of more than one previous neonatal deaths less than 20.

“+” means the association between history of more than one neonatal deaths and subsequent neonatal mortality is larger than the association between history of one neonatal death and subsequent neonatal mortality
